# Extemporaneously compounded liquid formulations of clofazimine

**DOI:** 10.5588/ijtld.22.0331

**Published:** 2023-02-01

**Authors:** R. Taneja, M. C. Nahata, J. Scarim, P. G. Pande, A. Scarim, G. Hoddinott, C. L. Fourie, R. K. Jew, H. S. Schaaf, A. C. Hesseling, A. J. Garcia-Prats

**Affiliations:** 1 Global Alliance for TB drug Development (TB Alliance), New York, NY, USA; 2 Institute of Therapeutic Innovations and Outcomes, Colleges of Pharmacy and Medicine, The Ohio State University, Columbus, OH, USA; 3 JSAS Services Inc, Tucson, AZ, USA; 4 Desmond Tutu TB Centre, Department of Paediatrics and Child Health, Faculty of Medicine and Health Sciences, Stellenbosch University, Cape Town, South Africa; 5 Metro TB Complex, Department of Health, Pretoria, South Africa; 6 Institute for Safe Medication Practices, Plymouth Meeting, PA, USA; 7 Department of Pediatrics, University of Wisconsin School of Medicine and Public Health, Madison, WI, USA

**Keywords:** anti-tuberculosis, medicine, suspension, stability

## Abstract

**BACKGROUND:**

Clofazimine (CFZ) is routinely used worldwide for the treatment of leprosy and TB. However, no liquid or dispersible tablet formulations of CFZ are currently available commercially for patients with challenges ingesting soft gelatin capsules or solid formulations. The aim of this research was to develop stable extemporaneous liquid formulations of CFZ that can be stored at room temperature for several weeks to enable practical dosing in the field.

**METHODS:**

Two formulations were prepared in syrup and sugar-free vehicles with CFZ tablets using a simple method that can be used in a routine pharmacy. Suspensions were stored at room temperature and at 30°C for 30 days. Formulation aliquots were tested on Days 0, 15 and 30 for appearance, pH, potency and microbial counts.

**RESULTS:**

Appearance remained unchanged during storage. The pH of both formulations was between 4.0 and 6.0. Potency was between 90% and 110% for 30 days in the syrup formulation and for 15 days in the sugar-free formulation. Microbial counts met United States Pharmacopeia <1111> limits for oral aqueous liquids and specific organisms were absent.

**CONCLUSIONS:**

A simple field-friendly method was successfully developed for the preparation of CFZ liquid formulations using commonly available ingredients. This will permit practical dosing and titration for children and other patients with swallowing challenges.

## Abstract (fr)

**CONTEXTE:**

La clofazimine (CFZ) est systématiquement utilisée dans le monde entier pour le traitement de la lèpre et de la TB. Toutefois, aucune préparation commerciale sous forme liquide ou sous forme de comprimés dispersibles de CFZ n’est actuellement disponible pour les patients qui ne peuvent avaler de gélules ou de préparations commerciales solides. L’objectif de cette étude était de développer des préparations liquides extemporanées stables de CFZ, pouvant se conserver à température ambiante pendant plusieurs semaines afin de faciliter le dosage sur le terrain.

**MÉTHODES:**

Deux suspensions des comprimés de CFZ ont été préparées avec et sans sucre, en utilisant une méthode simple qui peut être utilisée en pharmacie. Les suspensions ont été conservées à température ambiante et à 30°C pendant 30 jours. Les aliquotes ont été testées aux jours 0, 15 et 30 afin de déterminer l’apparence, le pH, la puissance et le taux de bactéries.

**RÉSULTATS:**

L’apparence est restée inchangée pendant la période de conservation. Le pH des deux préparations se situait entre 4,0 et 6,0. La puissance se situait entre 90% et 110% pendant 30 jours pour la préparation de sirop, et pendant 15 jours pour la préparation sans sucre. Le taux de bactéries était conforme aux seuils définis par la United States Pharmacopeia <1111> pour les formulations liquides aqueuses orales, et aucun organisme particulier n’a été retrouvé.

**CONCLUSIONS:**

Une méthode simple et facile à mettre en œuvre sur le terrain a été développée avec succès pour la préparation de formulations liquides de CFZ en utilisant des ingrédients couramment disponibles. Cette méthode permettra un dosage et une titration pratiques pour les enfants et les patients présentant des troubles de la déglutition.

Clofazimine (CFZ) is a broad-spectrum antimyco-bacterial agent recommended by the WHO as a first-line treatment for leprosy and as treatment for multidrug-resistant TB (MDR-TB).^1^ The WHO has included CFZ as part of Group B medicines recommended in longer MDR-TB regimens with at least four effective drugs.^2^

CFZ is a reddish-brown powder classified as a Biopharmaceutics Classification System (BCS) class II drug because of its poor aqueous solubility and high permeability. It is manufactured as soft gelatin capsules (Lamprene^®^; Novartis Pharmaceuticals Corp, East Hanover, NJ, USA ) in 50 and 100 mg strengths. The soft gelatin capsules (henceforth, ‘capsules’) contain micronized CFZ suspended in an oily base. CFZ capsules cannot be split, subdivided or triturated by utilizing a mortar and pestle. These formulations are not suitable for compounding or preparing extemporaneous formulations.

The lack of pediatric friendly dosing options for CFZ capsules has remained a challenge. It is highly problematic to administer capsules to young children who are unable to swallow them whole. Dose titration based on age or weight is also not feasible. In children, a dose of 2–5 mg/kg per day (maximum dose: 100 mg daily) is recommended.^3^ In the absence of other options, alternate day dosing of CFZ has been adopted for children to achieve the target daily dose on average because of the drug’s long half-life, however, there is limited evidence to support this approach.^4^ Walsh et al. also identified a major gap to dose young children and confirmed by clinicians’ experience, given the difficulty in manipulating the contents of the capsule.^5^ There is also a need for alternative formulations that meet the needs of patients with dysphagia or those who are intubated.

A review of literature did not yield any user-friendly method of preparing extemporaneous formulations of CFZ. In one instance, CFZ capsules had to be suspended in hot water and macerated with a hemostat prior to administration via a gastrostomy tube.^6^

There has been a description of flash nano-precipitated CFZ nanoparticles under development that could be dispersed in milk, but clinical evaluations have not been conducted.^7^ We have previously tried to extract the drug from the CFZ capsules by cutting them but could not perform a complete extraction of contents.

CFZ 50 mg and 100 mg tablets have recently been included in the list of WHO prequalified products for the treatment of TB. Bioequivalence of these tablets was established with reference to the Lamprene^®^ capsules.^8^

In this paper, we describe a method for the standard preparation of extemporaneously compounded suspensions of CFZ tablets that can be stored at room temperature for 2–4 weeks. These preparations were evaluated to ensure that the suspensions could be redispersed uniformly and the desired dose could be aliquoted accurately during the storage period. Microbial counts were also tested. These formulations would meet the needs for dosing children, intubated patients and patients suffering from dysphagia.

## METHODS

### Preparation of 10 mg/mL syrup formulation

Simple syrup was prepared by dissolving 255 g of cane sugar in 135 mL of hot distilled water by stirring. The syrup was allowed to cool to room temperature before use. Just prior to preparation of the extemporaneous formulation, the following ingredients were pre-measured: methylparaben powder 100 mg, potassium sorbate powder 100 mg, citric acid powder 125 mg, separate oral syringes with 10 mL of distilled water, 50 mL of simple syrup and 37 mL of simple syrup. Ten 100-mg CFZ tablets were ground into a fine powder in a glass mortar and pestle. The pre-weighed powders were added to the mortar and mixed with the ground tablets. Ten mL of distilled water was mixed with the powder using the pestle to make a smooth paste. Fifty mL syrup was added next and mixed for about 30 sec to make a uniform mixture; this was followed by the addition of the final 37 mL of the syrup. The preparation was mixed well to obtain 100 mL of a uniform CFZ 10 mg/mL suspension. The suspension was transferred into a 120-mL amber pharmacy dispensing bottle. A syringe adapter was inserted into the mouth of the bottle, followed by a child-resistant cap (CRC) to close the bottle. Stepwise preparation instructions are presented in Table 1.

**Table 1:** Instructions for preparation of CFZ 10 mg/mL formulation in simple syrup

Instructions for preparing 300 mL simple syrup (65% w/w) vehicle from cane sugar: Weigh 255 g of food-grade cane sugar into a suitable container Add 135 mL of hot distilled water and mix well until sugar is dissolved Cool syrup to ambient room temperature before use Instructions for 100 mL of CFZ 10 mg/mL formulation in simple syrup: Pre-weigh the following powders separately: methylparaben 100 mg, potassium sorbate 100 mg, citric acid 125 mg Measure out 10 mL of distilled water, 50 mL of simple syrup and 37 mL of simple syrup in separate oral syringes. Grind 10 tablets of CFZ (100 mg each) to a fine powder in a glass mortar and pestle Add the pre-weighed powders to the mortar and mix Mix powder with the 10 mL of distilled water to form a uniform paste Add the 50 mL of simple syrup and mix for 30–60 sec to obtain a uniform mixture Add the final 37 mL of syrup and mix well Transfer the contents from the mortar into an appropriately sized amber pharmacy dispensing bottle

w/w = weight by weight; CFZ = clofazimine.

### Preparation of 10 mg/mL sugar-free formulation

Thick & Easy^®^ (Hormel Foods Sales, Austin, MN, USA) sugar-free vehicle was prepared by stirring 8.9 g of Thick & Easy powder in 236 mL of distilled water for 30 sec. The vehicle was left undisturbed for 5 min and then stirred. The vehicle was mixed again prior to use. Just prior to preparation of the extemporaneous formulation, the following ingredients were pre-measured: methylparaben 100 mg, potassium sorbate 100 mg, citric acid 125 mg, sodium saccharin 125 mg, separate oral syringes with 50 mL of distilled water and 47 mL of Thick & Easy vehicle. Ten 100-mg CFZ tablets were ground into a fine powder in a glass mortar and pestle. The pre-weighed powders were added to the mortar and mixed with the ground tablets. The 50 mL of distilled water was mixed with the powder for 1–2 min using the pestle to obtain a smooth dispersion and dissolve the tablet coating; 47 mL of vehicle was added next and mixed for about 30–60 sec to make a uniform mixture. The preparation was mixed well to obtain 100 mL of a uniform CFZ 10 mg/mL suspension. The suspension was transferred into a 120-mL amber pharmacy dispensing bottle. A syringe adapter was inserted into the mouth of the bottle, followed by a CRC to close the bottle. Stepwise preparation instructions are presented in Table 2. A general technique for preparing the suspension and withdrawing doses is shown in the Figure.

**Figure:**
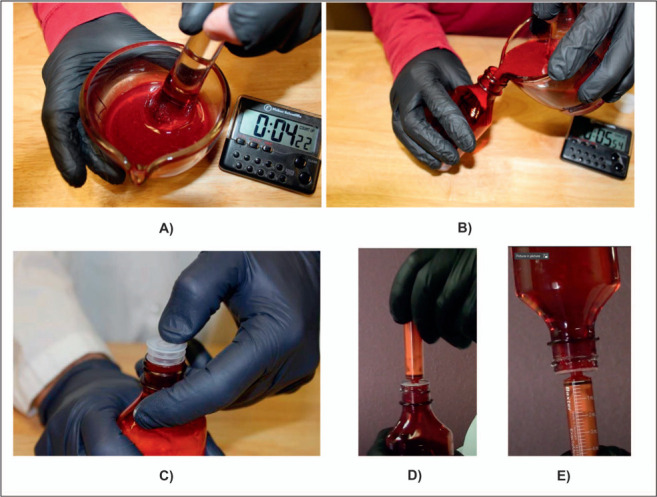
Preparation of CFZ suspensions: A) prepared CFZ suspension; B) transferring of CFZ suspension into a prescription bottle; C) inserting syringe adapter into mouth of bottle; D) inserting oral syringe into syringe adapter for dose withdrawal; and E) inverting bottle and withdrawal of dose aliquot for oral administration.

**Table 2:** Instructions for preparation of CFZ 10 mg/mL formulation in a sugar-free vehicle

Instructions for preparing 250 mL of Thick & Easy® sugar-free vehicle: Measure 236 mL (8 oz) of distilled water into a suitable container Add 8.9 g of Thick & Easy powder Mix well with a spoon for 30 seconds and let the mixture sit for at least 5 min before use Mix again just prior to use Instructions for 100 mL of CFZ 10 mg/mL sugar-free formulation: Pre-weigh the following powders separately: methylparaben 100 mg, potassium sorbate 100 mg, citric acid 125 mg, sodium saccharin 125 mg Measure out 50 mL of distilled water and 47 mL of Thick & Easy sugar-free vehicle in separate oral syringes Grind 10 tablets of CFZ (100 mg each) to a fine powder in a glass mortar and pestle Add the pre-weighed powders to the mortar and mix Add the 50 mL of distilled water into the mortar and mix for 1–2 min to obtain a uniform dispersion and dissolve any tablet coating 6 Add the 47 mL of Thick & Easy sugar-free vehicle and mix for 30–60 seconds to form a uniform suspension Transfer the contents from the mortar into an appropriately sized amber pharmacy dispensing bottle

CFZ = clofazimine.

### Storage for stability assessment

Twelve bottles of each formulation containing 100 mL were prepared as described above. Six bottles were stored for 30 days at ambient room temperature and six bottles were stored at 30°C/75% relative humidity (RH). For each storage condition, three bottles were used for potency testing, one bottle for appearance and pH, and one bottle for microbial testing. An extra bottle was stored as back up. Prior to withdrawal of samples, the bottles were shaken gently by inverting 30 times in 15 sec. Using a syringe attached to the syringe adapter, samples were withdrawn for the designated tests from each bottle on Days 0, 15 and 30.

### Appearance and pH

A 25-mL sample was poured from the bottle into a beaker. Visual appearance was recorded. A small amount was placed on a microscope slide and observed under a light microscope at 40X and 100X magnification. A calibrated pH meter was used to measure the pH of the sample in the beaker.

### Potency of clofazimine

The uniformity upon redispersion of the suspension at the storage timepoints was demonstrated by determining potency as follows: three bottles containing the formulation were stored at each condition. At each of the storage timepoints, the bottles were shaken and three 5-mL samples were withdrawn from the inverted bottles using a syringe connected to the syringe adapter on the mouth of the bottle (Figure). A total of nine samples per formulation at each timepoint were tested. The samples were analyzed using a stability-indicating high-performance liquid chromatography (HPLC) method.

### High-performance liquid chromatography method

A stability-indicating method was developed for determining the potency of CFZ in the formulations and verified for linearity, reproducibility, ruggedness, and specificity. Specificity was determined by forced degradation studies using CFZ drug substance. Forced degradation studies included exposure to light, heat, acid, base and oxidation. The HPLC system stability, system reproducibility, linearity, filter suitability and working standard stability were also determined.

Details of the HPLC method are provided in Table 3. Each 5-mL sample of the formulation was prepared for analysis by volumetrically diluting to 100 mL in the stock preparation diluent. A 4-mL aliquot of this sample solution was further diluted to 100 mL in working preparation diluent. An aliquot of this solution was filtered and analyzed.

**Table 3:** Details of stability-indicating HPLC method for potency of clofazimine formulations

Parameter	Details
Instrument	Hitachi L-2100 pump (Tokyo, Japan), Shimadzu (Kyoto, Japan) SPD-10AVP detector, Shimadzu SCL-10AVP controller, Shimadzu CTO-AVP column heater, SRI Instruments PeakSimple chromatography data system (Torrance, CA, USA)
Column	Waters Sunfire^®^ (Dresden, Germany) (C18, 4.6 mm × 100 mm, 3.5 μm)
Wavelength	283 nm
Column temperature	40°C
Flow rate	1.5 mL/min
Injection volume	10 μL
Syringe filter	Polypropylene, 0.45 μm
Stock standard concentration	0.5 mg/mL
Stock preparation diluent	Acetic acid:water: methanol (75:425:500)
Working standard concentration	0.02 mg/mL
Working preparation diluent	Water: acetonitrile (50:50)
Mobile phase	Water: acetonitrile: TFA (550:450:1)
Run time	8 min
Peak	6–7 min
Integration	Peak area method using PeakSimple data acquisition system, 5 Hz

HPLC = high-performance liquid chromatography; TFA = trifluoroacetic acid

### Evaluation of microbial growth

Suitability studies were conducted to evaluate and verify that the methods described in the United States Pharmacopeia (USP) <60>, USP<61>, and USP<62> were suitable to recover the specific microorganisms in the two CFZ formulations. The verified procedures were used to enumerate microbial counts (total aerobic microbial counts [TAMC] and total yeast and mold counts [TYMC]) and determine the absence of specific microorganisms in the two CFZ suspensions at Days 0, 15 and 30. The specific microorganisms tested in the formulations were *Staphylococcus aureus* (American Type Culture Collection [ATCC] 6538), *Pseudomonas aeruginosa* (ATCC9027), *Candida albicans* (ATCC10231), *Escherichia coli* (ATCC8739), *Aspergillus brasiliensis* (ATCC16404), *Burkholderia cepacia* (ATCC25416), and *Zygosaccharomyces rouxii* (ATCC28253), which included those tested with extemporaneous vehicles.^9^ One mL of sample from each CFZ suspension bottle was diluted with 9 mL of neutralizing broth. For positive and negative controls, 10 mL of neutralizing broth was utilized. The inoculum of each pathogen was prepared using phosphate buffer and serial dilution made with Tryptic Soy Broth [TSB] (Alpha Biosciences, Baltimore, MD, USA). A volume of 0.1 mL of the inoculum at a selected concentration of 10^3^ colony-forming units (cfu)/mL was spiked into the sample and the positive control tubes separately and mixed well. Negative control tubes were spiked with 0.1 mL of TSB and mixed well. One mL of the spiked sample, positive control, and negative control were placed on agar plates (bacteria on Tryptic Soy Agar [TSA] plates, and yeast and mold on Sabouraud Dextrose Agar [SDA] (Alpha Biosciences) plates). The TSA plates were incubated at 30–35°C for 3–5 days and SDA plates at 20–25°C for 5–7 days. The colonies for each microorganism were then counted and % recovery was calculated according to USP acceptance criteria (50–200%).

For specific microorganisms, appropriate plates were streaked and incubated per USP<62> at the specified temperatures for specified times, following pre-enrichment. After incubation, the presence or absence of the target organism was verified by comparing against positive controls.

### Outcome measures

The ease of suspension preparation was determined by noting the time required for completing the preparation. The consistency of the suspensions was determined based on the microscopic and visual appearance of the suspensions. The suspension pH was expected to be below 6.0 to ensure the effectiveness of the preservatives. The chemical and microbial stability was determined using standard testing procedures. The homogeneity and re-dispersibility upon shaking were determined on Days 0, 15 and 30 by testing the aliquots from the suspension. The formulations were homogeneous, and mean potency was acceptable if the measured CFZ concentrations of each of the 9 aliquots tested were within 10% of the theoretical value^10^ at each timepoint. The samples that met the USP<1111> limits for oral, aqueous liquid preparations in terms of TAMC and TYMC, and the absence of specified microorganisms tested in the formulations during the study period were considered acceptable.

### Ethics

As this was not a human subjects study, an ethics statement is not applicable to this study.

## RESULTS

It took less than 7 min to complete the preparation of the CFZ formulations. The suspensions could be redispersed with gentle inversion performed 30 times. Visual observation of the formulations showed that the formulations remained uniform and smooth at the specified time points throughout the duration of the storage. The appearance was smooth, uniform, dark purple to brown with no lumps or clumps of the tablet core. Microscopic observation of the formulations showed no change over the storage period. The pH ranged from 4.36 to 5.51 in the syrup formulation and from 4.93 to 5.67 in the sugar-free formulation over the duration of the 30-day storage.

As shown in Table 4, the mean potency across the 9 aliquots tested per timepoint over the duration of the stability fell within 90–110% of the theoretical value for both formulations; however, 1/3 individual values for two bottles for the Day 30 timepoint at room temperature and at 30°C in the sugar-free formulation were below 90% and failed to meet the potency criterion. Therefore, the sugar-free formulation was reported as stable for 15 days. The potencies of the syrup formulation ranged from 96.1% to 102.4% over the 30-day period. The potencies of the sugar-free formulation ranged from 98.0% to 102.3% over the 15-day period. To investigate the low values in one of the three aliquots withdrawn from each of two bottles of the sugar-free formulation, it was first confirmed that no degradation peaks were observed in the chromatograms. An aliquot was removed from the sugar-free formulation and spread onto a petri dish. Several small air bubbles were observed, indicating that air was getting entrapped as bubbles in the thick suspension during shaking of the potency samples for redispersion, resulting in lower potency values.

**Table 4:** Homogeneity of clofazimine suspension aliquots withdrawn during the 30-day storage period

Study day	Theoretical concentration remaining (*n* = 9)			

	Syrup formulation		Sugar-free formulation	
	
	RT	30°C	RT	30°C
	Mean % ± SD	Mean % ± SD	Mean % ± SD	Mean % ± SD
0	99.32 ± 1.07	99.33 ± 0.66	100.78 ± 1.04	100.57 ± 0.88
15	101.11 ± 0.89	100.89 ± 0.65	98.57 ± 0.93	97.68 ± 0.95
30	100.63 ± 1.06	100.23 ± 0.84	92.02 ± 2.26^*^	92.19 ± 2.72^*^

^*^ Of the three aliquots tested for potency from each of the three bottles, 1 out of 3 individual values for two bottles of sugar-free formulation failed to meet the acceptance criteria of 90–110% of theoretical concentration on Day 30. RT = room temperature; SD = standard deviation.

Microbial tests for the six specific microorganisms and *Burkholderia cepacia* complex showed no growth during the 30-day study period in either of the two formulations at room temperature and at 30°C. TAMC and TYMC met the USP<1111> limits for aqueous oral liquids during the entire 30-day study period in the syrup and sugar-free formulations (Table 5).

**Table 5:** Microbial limits test results of clofazimine suspensions during the 30-day storage period ^*^

Microorganism	Microbial limits test results					

	Syrup formulation (RT and 30°C)			Sugar-free formulation (RT and 30°C)		
	
	Day 0 cfu/g	Day 15 cfu/g	Day 30 cfu/g	Day 0 cfu/g	Day 15 cfu/g	Day 30 cfu/g
TAMC	<10	<10	<10	<10	<10	<10
TYMC	<10	<10	<10	<10	<10	<10

^*^ Per USP<1111>, acceptable criteria for microbiological quality of oral aqueous liquids are TAMC <200 cfu/g and TYMC <20 cfu/g, and absence of *E. coli*.

RT = room temperature; cfu = colony-forming unit; TAMC = total aerobic microbial counts; TYMC = total yeast and mold counts; USP = United States Pharmacopeia.

CFZ is a red dye and extra caution is required during the crushing and dispersion of the tablets. The preparation imparts a red color to the equipment and any surface in contact. The final preparations were stored in amber colored pharmacy dispensing bottles to prevent any photodegradation.

## DISCUSSION

CFZ 10 mg/mL formulations in syrup and sugar-free vehicles were easily prepared using ingredients that are commonly available and easy to source. Overall, the chemical stability of the syrup and sugar-free formulations met the pre-established criteria of 90–110% of the potency. Potency of the nine samples were in close agreement, indicating that the redispersed suspensions were uniform. The sugar-free formulations met the homogeneity criteria at the 15-day evaluation but did not meet the homogeneity criteria at the 30-day evaluation point. As mentioned earlier, we wanted to make these field-friendly formulations by utilizing readily available simple excipients. Adding different but less readily available suspending vehicles could potentially have made the sugar-free suspension homogenously re-dispersible for a longer duration.

We followed the American Society of Health-System Pharmacists (Bethesda, MD, USA)^11^ and USP^12^ guidelines for compounded aqueous nonsterile preparations. Selection of preservatives were based on the recommended concentrations from literature^13^ and commercially available compounding vehicles.^14^ This has been the standard approach in published stability studies of oral liquids for use in pediatric patients in peer-reviewed pharmacy journals. We conducted the stability studies for 30 days to meet the beyond-use date (BUD) requirements for USP<795>.^15^ The assigned BUD was confirmed using stability testing and microbial count/absence of specific organisms.

The recently introduced 50- and 100-mg CFZ tablets provide a convenient field-friendly option for making extemporaneous formulations. These tablets can be easily crushed and dispersed in liquid media. We intentionally used easily accessible and affordable ingredients (e.g., cane sugar, Thick & Easy) to prepare the formulations to ensure relevance of our methods in countries where CFZ are likely to be used. Cane sugar can be substituted with powdered sugar. Simple syrup, if commercially available, can be used as an alternative. Organoleptic properties can be enhanced by utilizing additional flavors and sweeteners, and conducting appropriate stability studies.

## CONCLUSIONS

We have demonstrated simple field-friendly methods for preparing syrup and sugar-free extemporaneous suspension formulations of CFZ. These formulations allow for more ready administration and accurate dose titration of CFZ in children and other patients. These suspensions are easy to prepare using commonly available excipients and may improve dosing of CFZ, thereby reducing complexities in administering treatment.
